# An Initial Examination of Couple Therapy for PTSD Outcomes Among Black/African American Adults: Findings from an Uncontrolled Trial with Military Dyads

**DOI:** 10.3390/bs15040537

**Published:** 2025-04-16

**Authors:** Steffany J. Fredman, Alyssa A. Gamaldo, August I. C. Jenkins, Yunying Le, Jacqueline A. Mogle, Candice M. Monson, Charlene E. Gamaldo, Roland J. Thorpe, Brittany N. Hall-Clark, Tabatha H. Blount, Brooke A. Fina, Orfeu M. Buxton, Christopher G. Engeland, Galena K. Rhoades, Scott M. Stanley, Alexandra Macdonald, Katherine A. Dondanville, Daniel J. Taylor, Kristi E. Pruiksma, Brett T. Litz, Stacey Young-McCaughan, Jeffrey S. Yarvis, Terence M. Keane, Alan L. Peterson

**Affiliations:** 1 Department of Human Development and Family Studies, The Pennsylvania State University, University Park, PA 16802, USA; 2 Department of Psychology, Clemson University, Clemson, SC 29634, USA; agamald@clemson.edu (A.A.G.);; 3 Department of Human Development and Family Studies, University of Illinois Urbana-Champaign, Urbana, IL 61801, USA; aij0006@auburn.edu; 4 Department of Human Development and Family Science, Auburn University, Auburn, AL 36849, USA; 5 Department of Psychology, University of Denver, Denver, CO 80210, USA; yunying.le@du.edu (Y.L.); grhoades@du.edu (G.K.R.); scott.stanley@du.edu (S.M.S.); 6 Department of Psychology, Toronto Metropolitan University, Toronto, ON M5B 2K3, Canada; candice.monson@torontomu.ca; 7 Department of Neurology, Johns Hopkins School of Medicine, Baltimore, MD 21287, USA; cgamald1@jhmi.edu; 8 Department of Health, Behavior and Society, Johns Hopkins Bloomberg School of Public Health, Baltimore, MD 21202, USA; rthorpe@jhu.edu; 9 Department of Psychiatry and Behavioral Sciences, The University of Texas Health Science Center at San Antonio, San Antonio, TX 78229, USA; hallclark@uthscsa.edu (B.N.H.-C.); blountt@uthscsa.edu (T.H.B.); fina@uthscsa.edu (B.A.F.); dondanville@uthscsa.edu (K.A.D.); pruiksma@uthscsa.edu (K.E.P.); youngs1@uthscsa.edu (S.Y.-M.); petersona3@uthscsa.edu (A.L.P.); 10 Department of Biobehavioral Health, The Pennsylvania State University, University Park, PA 16802, USA; orfeu@psu.edu (O.M.B.); cge2@psu.edu (C.G.E.); 11 Ross and Carole Nese College of Nursing, The Pennsylvania State University, University Park, PA 16802, USA; 12 Department of Psychology, The Citadel, Military College of South Carolina, Charleston, SC 29409, USA; amacdon1@citadel.edu; 13 Department of Psychology, University of Arizona, Tucson, AZ 85721, USA; danieljtaylor@arizona.edu; 14 Massachusetts Veterans Epidemiology Research and Information Center, VA Boston Healthcare System, Boston, MA 02130, USA; litzb@bu.edu; 15 Department of Psychiatry, Boston University School of Medicine, Boston, MA 02118, USA; terry.keane@va.gov; 16 Research and Development Service, South Texas Veterans Health Care System, San Antonio, TX 78229, USA; 17 Department of Behavioral Health, Carl R. Darnall Army Medical Center, Fort Cavazos, TX 76543, USA; jyarvis@tulane.edu; 18 Behavioral Science Division, National Center for PTSD, VA Boston Healthcare System, Boston, MA 02130, USA; 19 Department of Psychology, The University of Texas at San Antonio, San Antonio, TX 78249, USA

**Keywords:** Black, African American, posttraumatic stress disorder, PTSD, couples, couple therapy, military, veteran, treatment, retreat

## Abstract

Black/African American individuals experience high rates of posttraumatic stress disorder (PTSD), which is frequently chronic and undertreated in this population. Intimate relationships are a salient resource for Black/African American adults’ psychological well-being. To help advance health equity, this study serves as an initial, proof-of-concept investigation of patient outcomes among Black/African American adults who received a disorder-specific couple therapy for PTSD. Participants were a subsample of seven Black/African American adults (mean age = 40.56 years, *SD* = 10.18; 85.7% male) who participated in an uncontrolled trial of an abbreviated, intensive, multi-couple group version of cognitive-behavioral conjoint therapy for PTSD with 24 military dyads. Treatment was delivered over 2 days in a weekend retreat format. Assessments were administered at baseline, 1 month post-retreat, and 3 months post-retreat. There were large and significant decreases in patients’ PTSD symptoms based on clinicians’ and patients’ ratings (*d*s −1.37 and −1.36, respectively) by the 3-month follow-up relative to baseline. There were also large and significant decreases in patients’ depressive, anxiety, and anger symptoms (*d*s −1.39 to −1.93) and a large, marginally significant decrease in patients’ insomnia (*d* = −0.85; *p* = 0.083). Patients reported a medium, non-significant increase in relationship satisfaction (*d* = 0.68; *p* = 0.146) and a large, marginally significant increase in joint dyadic coping (*d* = 0.90; *p* = 0.069). Findings offer preliminary evidence that treating PTSD within a couple context is a relevant strategy to reduce PTSD and comorbid symptoms among partnered Black/African American adults and a promising approach to enhance relationships.

## 1. Introduction

The lifetime prevalence of posttraumatic stress disorder (PTSD; APA, 2013) among Black/African American individuals is high, with estimates ranging from 9 to 33% (Alim et al., 2006; Roberts et al., 2011). Among Black/African American individuals, PTSD also tends to be chronic (Sibrava et al., 2019) and undertreated (Nobles et al., 2016; Roberts et al., 2011). Research underscores the role of social disadvantage in Black/African American individuals’ trauma exposure, high rates of PTSD, chronicity of PTSD, and undertreatment of PTSD (MacIntyre et al., 2023; Sibrava et al., 2019; Torres et al., 2024). Family relationships are salient in Black/African American adults’ ability to cope with stress and trauma (Kelly et al., 2020; McNeil Smith & Landor, 2018; Nguyen et al., 2016), with intimate relationships serving as a resource for psychological health and well-being (Lincoln & Chae, 2010; Taylor et al., 2001, 2012). Given that intimate relationships play an important role in Black/African American individuals’ mental health, treating PTSD using a couple-based approach may be a clinically and culturally relevant strategy for promoting health equity in this population. Cognitive-behavioral conjoint therapy for PTSD (CBCT for PTSD; Monson & Fredman, 2012) has demonstrated efficacy in simultaneously reducing PTSD and enhancing relationship functioning (e.g., Monson et al., 2012; Morland et al., 2022). However, to our knowledge, there are no studies examining CBCT for PTSD or other disorder-specific couple/family-based treatments for PTSD among Black/African American individuals.

This study investigated outcomes within a racially homogeneous subsample of Black/African American patients who participated in a larger uncontrolled trial of an abbreviated, intensive, multi-couple group version of CBCT for PTSD (Fredman et al., 2020) delivered in a workshop-style weekend retreat for active duty military and veteran couples. All participants in the parent study (*N* = 24 couples) completed treatment (i.e., 0% dropout), and, by 3-month follow-up, there were large and significant reductions in clinicians’ and patients’ ratings of patients’ PTSD symptoms (*d*s −0.98 and −1.17, respectively) and in patients’ self-reported depressive, anxiety, and anger symptoms (*d*s −0.60 to −0.75). There were also significant increases in patients’ perceptions of joint dyadic coping (i.e., working effectively as a couple when both dyad members are stressed; *d* = 0.57). Changes in patients’ insomnia (*d* = −0.34) and relationship satisfaction (*d* = 0.10) were small and nonsignificant (Fredman et al., 2020, 2021; Macdonald et al., 2022). Although the parent study showed promising results, the larger sample was racially heterogeneous, potentially masking distinctive patterns of response within a racial group (Whitfield et al., 2008). Thus, this secondary analysis is an initial investigation of the magnitude of effects among patients identifying as Black/African American.

## 2. Materials and Methods

### 2.1. Participants

The sample for the current study consisted of seven patients from a previously published clinical trial (Fredman et al., 2020), with all patients and their intimate partners in the current study self-identifying as Black/African American. The parent study was conducted as part of the Consortium to Alleviate PTSD (Peterson et al., 2021), a multi-disciplinary, multi-institutional research consortium focused on the study, prevention, and treatment of PTSD and related conditions in active duty service members and veterans. Patients in the parent study were U.S. service members or veterans who (a) met diagnostic criteria for PTSD according to the *Diagnostic and Statistical Manual of Mental Disorders* (5th ed.; APA, 2013) as determined by the Clinician-Administered PTSD Scale for *DSM-5* (CAPS-5; Weathers, Blake, et al., 2013), (b) had a minimum CAPS-5 severity score of 25, and (c) had experienced a Criterion A traumatic event during deployment in support of combat operations following 11 September 2001, although the index traumatic event that served as the basis for diagnosing PTSD did not need to be combat-related (e.g., child abuse, sexual assault). Couples were required to be married or cohabiting for a minimum of 3 months, 18–65 years of age, and able to speak, read, and write fluently in English. A full description of study inclusion and exclusion criteria is included in Fredman et al. (2020).

Similar to the parent study, most patients in the current study identified as male and had a female partner (*n* = 6, 85.7%). One couple consisted of a female patient with a male partner. As in the parent study, patients were approximately 40 years of age (mean = 40.56 years; *SD* = 10.18). Mean relationship length for the current analytic sample was 15.13 years (*SD* = 10.53; range = 1–31 years). Most patients were on active duty (*n* = 5, 71.4%) and were enlisted (*n* = 6, 85.7%). A complete description of baseline characteristics of patients and study procedures for the parent study can be found in Fredman et al. (2020).

### 2.2. Procedure

Both members of the couple provided written informed consent. Independent evaluators conducted assessments at baseline and 1 and 3 months after the retreat. All study procedures were approved by the Institutional Review Board (IRB) at The University of Texas Health Science Center at San Antonio (UT Health San Antonio) and the Research Ethics Board at Ryerson University (now Toronto Metropolitan University). All other IRBs for institutions with which investigators were affiliated deferred their review to the IRB at UT Health San Antonio or were considered exempt. The U.S. Army Medical Research and Materiel Command Human Research Protections Office (now the U.S. Army Medical Research and Development Command) at Fort Detrick, Maryland, oversaw regulatory reviews and approvals. This secondary analysis was not preregistered. Study data, materials, and code for the analyses are maintained at UT Health San Antonio in the STRONG STAR Repository. Requests for data can be emailed to repository@strongstar.org.

#### Treatment Protocol

The intervention was an abbreviated, intensive, multi-couple group version of CBCT for PTSD (Fredman et al., 2020; Monson & Fredman, 2012) delivered over 2 consecutive days during a single weekend retreat in Austin, Texas. Each couple also participated in two meetings; one meeting occurred 1–2 weeks before the retreat to orient them to treatment, and one occurred approximately 2 weeks after the retreat to reinforce ongoing skill use. Approximately 12 h of CBCT for PTSD content was delivered using a workshop format. Content included (a) psychoeducation about the bidirectional linkages between PTSD and intimate relationship adjustment, (b) conflict management, communication, and problem-solving skills to enhance relationship adjustment and reduce PTSD-related avoidance and symptom accommodation, and (c) dyadic cognitive interventions to promote flexibility in both couple members’ thinking about the trauma and its effects on couples’ relationships. On the Saturday evening of the retreat, couples went on a date that also served as a PTSD-relevant approach (i.e., exposure) activity. From 2016 to 2017, seven retreats were conducted, and these ranged in size from two to six couples. For more details about the intervention and fidelity monitoring, see Fredman et al. (2020).

### 2.3. Measures

Patients’ PTSD symptoms were assessed using the CAPS-5 (Weathers, Blake, et al., 2013), a gold standard diagnostic interview for determining PTSD diagnosis and symptom severity. The CAPS-5 consists of 20 items that correspond to the *DSM-5* PTSD symptoms in the past month. Across the Consortium’s studies, interrater reliability for the CAPS-5 was excellent regarding both presence/absence of PTSD (Cohen’s ĸ = 0.90) and concordance of PTSD symptom severity scores between independent evaluators and an expert rater (*r* = 0.98; Barnes et al., 2019). All other measures were based on patient self-report. Patient-rated PTSD symptom severity was assessed via the PTSD Checklist for *DSM-5* (Weathers, Litz, et al., 2013). Depressive symptoms were assessed via the Patient Health Questionnaire-9 (Kroenke et al., 2001), generalized anxiety symptoms via the Generalized Anxiety Disorder Screener (Spitzer et al., 2006), feelings of anger at a given moment via the State Anger subscale of the State-Trait Anger Expression Inventory-2 (Spielberger, 1999), and insomnia symptoms via the Insomnia Severity Index (Morin, 1993). Relationship satisfaction was assessed with the 32-item Couples Satisfaction Index (Funk & Rogge, 2007); joint dyadic coping was assessed with the Joint Dyadic Coping subscale of the Dyadic Coping Inventory (Bodenmann, 2008). As reported previously (Fredman et al., 2020, 2021; Macdonald et al., 2022), across assessments, internal consistency was satisfactory for all measures in the parent study (α = 0.76 to 0.90).

### 2.4. Data Analysis

Multilevel models with time classified as a categorical variable were conducted using PROC MIXED in SAS^®^ OnDemand for Academics using data from the baseline, 1-month follow-up, and 3-month follow-up assessments. For the model predicting changes in self-reported PTSD symptoms, data from the post-retreat check-in were also used. Models employed robust standard errors and restricted maximum likelihood estimation. An unstructured covariance matrix was used to freely estimate variances and covariances across the repeated measures. To determine whether there were statistically significant changes in outcomes for each assessment relative to pretreatment levels on average across patients, planned contrasts between pretreatment and 3-month post-treatment least squares means generated from the multilevel models were conducted. Within-group effect sizes in the form of Cohen’s *d* ($\frac{t}{\sqrt{df}}$) were computed to determine the magnitude of change in outcomes from pretreatment to 3-month follow-up, the endpoint of interest. Effect sizes were interpreted consistently with Cohen’s (1988) recommendations for small (*d* = 0.20), medium (*d* = 0.50), and large (*d* = 0.80) effect sizes. At the individual level, reliable change was examined for each patient’s clinician- and patient-rated PTSD symptoms following procedures outlined by Jacobson and Truax (1991) and used in Fredman et al. (2020). In the current sample, six of seven patients (85.7%) provided 1- and 3-month follow-up data, and all patients provided self-reported PTSD symptom data on the PCL-5 at the post-retreat check-in. The CONSORT chart in Fredman et al. (2020) includes details about the flow of participants in the parent study.

## 3. Results

Least squares means and their standard errors at each assessment point, *t* statistics (and associated *p* values), and Cohen’s *d*s with 95% confidence intervals for planned contrasts between pretreatment and 3-month follow-up levels for each outcome are reported in Table 1.

From pretreatment to 3-month follow-up, there were large and significant reductions in patients’ PTSD symptoms according to both independent evaluator and patient ratings (*d*s = −1.37 and −1.36, respectively), as well as in patients’ self-reported depressive symptoms (*d* = −1.87), anxiety (*d* = −1.93), and anger (*d* = −1.39). A large, marginally significant reduction in insomnia (*d* = −0.85; *p* = 0.083) was also observed. Patients reported a medium, non-significant increase in relationship satisfaction (*d* = 0.68; *p* = 0.146) and a large, marginally significant increase in joint dyadic coping (*d* = 0.90; *p* = 0.069).

Two thirds of patients who completed the 3-month follow-up assessment (four of six; 66.7%) were classified as reliably improved according to independent evaluators’ ratings on the CAPS-5; similarly, 66.7% were classified as reliably improved based on their self-rated symptoms on the PCL-5. No patients were classified as reliably worsened.

## 4. Discussion

To our knowledge, this is the first study to investigate outcomes for Black/African American adults participating in couple therapy for PTSD. Findings from this small subsample of Black/African American active duty military and veteran patients who participated in an abbreviated, multi-couple group version of CBCT for PTSD provide preliminary support for the notion that treating PTSD within a couple context may be an effective and culturally relevant strategy to reduce PTSD and comorbid symptoms among partnered Black/African American adults. There were also promising trends for sleep and relationship functioning, which collectively have important public health implications.

Notably, this treatment targeted the intersection between PTSD and relationship adjustment and did not include an explicit focus on sleep, yet large decreases in insomnia were still observed. The trend of large reductions in insomnia within this small subsample of Black/African American adults (*d* = −0.85) but not in the full, racially mixed sample (*d* = −0.34) supports further exploration of this promising signal in a larger sample of Black/African American individuals. These disparate effect size findings observed between the two research approaches (i.e., exploration within a Black/African American sample versus within a racially mixed sample) support prior literature (e.g., Whitfield et al., 2008) that has suggested that the within-racial-group approach used in the current study can provide meaningful information that may otherwise be obscured. The current study’s preliminary findings regarding reductions in insomnia among Black/African American adults are also important from a public health perspective given that poor sleep is a mechanism contributing to myriad health disparities among Black/African American adults (Jackson et al., 2020). Findings from a large, nonclinical sample of rural Black/African American couples (Barton et al., 2021) demonstrated that a couple-based relationship education program exerted improvements in participants’ mental health, problematic sleep, and general health through improvements in couple functioning. Thus, future work that delivers PTSD treatment in a couple-based format may be a fruitful strategy to advance health equity broadly, having the potential to impact psychological, relational, and behavioral/physical health domains at the same time.

There are several possible reasons for the large effects observed across multiple outcomes in this subsample. First, the treatment was delivered in a couple context. As such, the involvement of patients’ spouses as an integral part of the treatment may have felt particularly supportive and relevant. The emphasis on interdependence between patients and partners in the context of a shared stressor (i.e., PTSD), as well as the recurrent theme of addressing PTSD as a couple throughout the treatment, may also have resonated with patients given the high levels of collectivistic coping orientation among many Black/African American individuals and families (McNeil Smith & Landor, 2018). Relatedly, delivering treatment in a multi-couple group format may have been salient for patients given its alignment with Afro-centric values around collectivism and communalism—ideals that promote the success of the individual and the group as being intertwined (Johnson & Carter, 2020).

This proof-of-concept study is novel in its research question, patient population, and findings. There are also limitations. The uncontrolled nature of the parent study precludes the ability to attribute changes in outcomes to the intervention specifically, as these changes could be due to regression to the mean, natural recovery, and/or nonspecific therapeutic factors. Another limitation is the small sample size, which may have limited power to reach statistical significance for some effects that were medium (e.g., increases in relationship satisfaction; *d* = 0.68; *p* = 0.146) or large in size, such as the reductions in insomnia (*d* = −0.85; *p* = 0.083) and increases in perceived joint dyadic coping (*d* = 0.90; *p* = 0.069). The small sample size could have also increased the risk for Type I error and overestimated the magnitude of observed effects (Button et al., 2013). Additionally, nearly 90% of patients in this secondary analysis were Black/African American men with female partners, yet the lifetime prevalence of PTSD in Black/African American women is more than twice that of Black/African American men (Valentine et al., 2019). Providing for one’s family instrumentally and emotionally is central to the identity and values of many married Black/African American men (Hurt et al., 2017). Thus, in this context, working with their partners to decrease the salience of PTSD in their relationships in the service of experiencing more loving and satisfying romantic and broader family relationships may have been especially reinforcing for men. That said, we are not aware of any reasons that the treatment would not work equally well for Black/African American women.

To increase confidence in the findings, as well as to better understand PTSD and its treatment at various social intersections, future studies should test the efficacy of CBCT for PTSD using a randomized controlled design with a racially homogeneous sample of Black/African American couples that is larger, includes more female-identified patients, and is more diverse regarding patient gender, gender composition of couples, and couples’ military versus civilian status. In addition, research in this area that includes coparenting and parenting outcomes would be culturally relevant (as collective investment in effectively parenting children to navigate a racialized society is a noted strength of Black/African American families; Littlejohn-Blake & Darling, 1993) and may provide further evidence of the relational impacts of this treatment. Given the heterogeneity among Black/African American individuals and families in terms of their experiences with environmental stressors (e.g., interpersonal and structural racism) and access to resources (e.g., racial identity, religion/spirituality, extended kin networks; Kelly et al., 2020; McNeil Smith & Landor, 2018), future studies should also examine socio-ecological characteristics that may moderate treatment outcomes, as this may help to inform a comprehensive, patient-centered approach to treatment as part of advancing health equity (Lau et al., 2023; Purnell et al., 2016).

Findings from this small proof-of-concept study are encouraging with respect to the use of CBCT for PTSD for treating PTSD and comorbid symptoms and enhancing intimate relationship functioning among Black/African American adults with PTSD. Delivering treatment in a compressed, multi-couple group format may confer additional benefits given its efficiency and use of the collective as a vehicle for stimulating recovery from the disorder. Additional research and investment in the use of culturally relevant, disorder-specific couple therapies for PTSD for Black/African American adults offers the potential to enhance mental and relational health equity for this population and others experiencing elevated rates of posttraumatic stress symptomatology.

## Figures and Tables

**Table 1:** Pre- and post-treatment outcomes for abbreviated, intensive, multi-couple group cognitive-behavioral conjoint therapy for posttraumatic stress disorder (PTSD) for patients identifying as Black or African American.

Outcome	Least Squares Mean (Standard Error)			Pretreatment/3-Month Post-Treatment		
	Pretreatment	1-Month Post-Treatment	3-Months Post-Treatment	*t* (6)	*p*	*d* (95% CI)
CAPS-5	33.57 (2.78)	21.18 (3.76)	21.99 (3.83)	−3.35 *	0.015	−1.37 (−0.37, −2.37)
PCL-5	52.71 (4.88)	28.39 (4.62)	32.08 (3.95)	−3.32 *	0.016	−1.36 (−0.36, −2.35)
PHQ-9	17.43 (1.56)	11.31 (1.79)	9.80 (1.06)	−4.58 *	0.004	−1.87 (−0.87, −2.87)
GAD-7	14.86 (1.10)	10.40 (1.89)	8.26 (2.10)	−4.72 *	0.003	−1.93 (−0.93, −2.93)
STAXI-2	29.86 (3.41)	18.60 (0.97)	19.65 (1.70)	−3.41 *	0.014	−1.39 (−0.39, −2.39)
ISI	22.00 (1.77)	19.86 (1.52)	16.37 (2.61)	−2.08 †	0.083	−0.85 (0.15, −1.85)
CSI	109.00 (15.26)	131.31 (6.71)	125.46 (8.14)	1.67	0.146	0.68 (−0.32, 1.68)
JDC	14.57 (2.35)	17.02 (1.69)	17.75 (1.72)	2.21 †	0.069	0.90 (−0.10, 1.90)

Note. *N* = 7. CI = confidence interval; CAPS-5 = Clinician-Administered PTSD Scale for *DSM-5*; PCL-5 = PTSD Checklist for *DSM-5*; PHQ-9 = Patient Health Questionnaire-9; GAD-7 = Generalized Anxiety Disorder Screener; STAXI-2 = State Trait Anger Expression Inventory-2 State Subscale; ISI = Insomnia Severity Index; CSI = Couples Satisfaction Index; JDC = Joint Dyadic Coping subscale of the Dyadic Coping Inventory. At 2-week follow-up, the least squares mean for PCL-5 for patients = 41.57 (4.94). † *p* < 0.10, * *p* < 0.05.

## Data Availability

Study data are maintained at The University of Texas Health Science Center at San Antonio in the STRONG STAR Repository. Requests for access to the data can be emailed to repository@strongstar.org.
